# Very high intact-protein formula successfully provides protein intake according to nutritional recommendations in overweight critically ill patients: a double-blind randomized trial

**DOI:** 10.1186/s13054-018-2070-5

**Published:** 2018-06-12

**Authors:** Arthur R. H. van Zanten, Laurent Petit, Jan De Waele, Hans Kieft, Janneke de Wilde, Peter van Horssen, Marianne Klebach, Zandrie Hofman

**Affiliations:** 1 Department of Intensive Care Medicine, Gelderse Vallei Hospital, Ede, The Netherlands; 2 Surgical and Trauma Intensive Care Unit, Pellegrin University Hospital, Bordeaux, France; 3 Department of Critical Care Medicine, Ghent University Hospital, Ghent, Belgium; 4 Department of Intensive Care, Isala Hospital, Zwolle, The Netherlands; 5 Nutricia Research, Utrecht, The Netherlands

**Keywords:** ICU, Enteral nutrition, High protein, Nutritional guidelines, Intact protein

## Abstract

**Background:**

Optimal energy and protein provision through enteral nutrition is essential for critically ill patients. However, in clinical practice, the intake achieved is often far below the recommended targets. Because no polymeric formula with sufficient protein content is available, adequate protein intake can be achieved only by supplemental amino acids or semi-elemental formula administration. In the present study, we investigated whether protein intake can be increased with a new, very high intact-protein formula (VHPF) for enteral feeding.

**Methods:**

In this randomized, controlled, double-blind, multicenter trial, 44 overweight (body mass index ≥ 25 kg/m^2^) intensive care unit patients received either a VHPF (8 g/100 kcal) or a commercially available standard high protein formula (SHPF) (5 g/100 kcal). Protein and energy intake, gastrointestinal tolerance (gastric residual volume, vomiting, diarrhea, and constipation), adverse events, and serious adverse events were recorded. Total serum amino acid levels were measured at baseline and day 5.

**Results:**

The primary outcome, protein intake at day 5, was 1.49 g/kg body weight (95% CI 1.21–1.78) and 0.76 g/kg body weight (95% CI 0.49–1.03, *P* < 0.001) for VHPF and SHPF, respectively. Daily protein intake was statistically significantly higher in the VHPF group compared with the SHPF group from day 2 to day 10. Protein intake in the VHPF group as a percentage of target (1.5 g/kg ideal body weight) was 74.7% (IQR 53.2–87.6%) and 111.6% (IQR 51.7–130.7%) during days 1–3 and days 4–10, respectively. Serum amino acid concentrations were higher at day 5 in the VHPF group than in the SHPF group (*P* = 0.031). No differences were found in energy intake, measures of gastrointestinal tolerance, and safety.

**Conclusions:**

Enteral feeding with VHPF (8 g/100 kcal) resulted in higher protein intake and plasma amino acid concentrations than an isocaloric SHPF (5 g/100 kcal), without an increase in energy intake. This VHPF facilitates feeding according to nutritional guidelines and is suitable as a first-line nutritional treatment for critically ill overweight patients.

**Trial registration:**

Netherlands Trial Register, NTR5643. Registered on 2 February 2016.

**Electronic supplementary material:**

The online version of this article (10.1186/s13054-018-2070-5) contains supplementary material, which is available to authorized users.

## Background

Extensive loss of total body protein mass is a universally observed phenomenon during critical illness. Breakdown of skeletal muscles is the primary source of amino acids for the synthesis of acute-phase proteins and immunoglobulins. Loss of muscle protein occurs rapidly in critically ill patients and may result in a loss of up to 18% of muscle mass in the first 10 days of intensive care unit (ICU) stay, leading to a marked negative total body nitrogen balance. The magnitude of protein loss has been associated with increased morbidity and mortality [1, 2]. Sufficient supply of proteins is considered essential to preventing protein energy malnutrition and may improve clinical outcomes [3, 4].

Several observational studies have demonstrated that a higher intake of enteral nutrition was associated with lower mortality rates [5–7]. More recent data indicate that better mortality reduction is achieved by meeting both caloric and protein targets than by meeting only caloric targets [8]. In addition, some recent reports suggest that lower caloric intake (i.e., 70–90% of energy expenditure) may be optimal in the initial phase of critical illness, whereas caloric overfeeding is associated with a worse outcome [9, 10]. In contrast, increased protein intake was associated with a linear dose-dependent decrease in mortality rate [10]. On the basis of these studies, the current consensus is to provide adequate protein intake as a primary goal [11].

A protein goal of 1.5 g/kg body weight (BW) is commonly recommended [12, 13]. The American Society of Enteral and Parenteral Nutrition and the International Protein Summit 2016 recommend a minimum protein intake of 1.2 g/kg BW, increasing to 2.0 to 2.5 g/kg ideal body weight (IBW) per day in obese patients [11]. Based on observational and nitrogen balance studies, the optimal intake may also be at the higher end of this range in elderly, burn, multitrauma patients or patients receiving continuous renal replacement therapy [1, 11]. Cumulative increases in nitrogen balance were found with protein dosages up to 2 g/kg BW without an increase in oxidation [14]. Other investigators have suggested even higher dosages to improve outcome, regardless of total body protein balance [15].

Despite these widely accepted guidelines, a large gap remains between the amounts recommended by nutrition authorities and actual protein provision in clinical practice. Observational studies have reported mean protein intakes of around 0.7 g/kg BW/d [6, 16] and few patients reaching the minimum 1.2 g/kg BW target [8, 17]. Such low delivery of protein is caused in part by known barriers to enteral nutrition administration, including gastrointestinal intolerance and interruptions for other procedures. In addition, the relatively low protein content in commercially available enteral nutrition formulas makes it challenging to achieve recommended protein targets. Options such as parenteral amino acids or enteral protein supplements are available to increase protein intake without caloric overfeeding, but they do not seem to be commonly applied, and monitoring protein adequacy in addition to caloric intake is far from standard practice. Therefore, several authors have highlighted the need to develop solutions to provide more proteins, such as high or very high protein formulas (VHPFs) for enteral feeding [1, 11, 18].

Recently, formulas with high protein content based on hydrolyzed protein became available. However, nutrition societies still recommend standard whole protein (e.g., intact protein or polymeric) feeds as first-line treatment for the majority of ICU patients because no advantage has been shown for hydrolyzed formulas [11]. To enable protein provision according to the recommended daily amounts (1.2–2.5 g/kg BW) with a polymeric formula, an intact -protein product was developed with an increased protein-to-energy ratio.

The aim of this study was to investigate protein and energy intake, gastrointestinal tolerance, and safety of this new polymeric VHPF. We hypothesized that a higher protein intake can be achieved with a VHPF than with a standard high protein formula (SHPF) without increasing energy intake and gastrointestinal problems.

## Methods

### Study design

We conducted a multicenter, randomized, controlled, double-blind, parallel-group study. The study was performed in four centers in three European countries: The Netherlands (two centers), France (one center), and Belgium (one center). The study protocol was approved by institutional review boards at each location and registered with the Netherlands Trial Register with the identifier NTR5643 (http://www.trialregister.nl/trialreg). Study procedures were performed in accordance with the Declaration of Helsinki ethical principles for medical research involving human subjects. Written informed consent was obtained from patients or their legal representatives.

The study population comprised mechanically ventilated patients (aged ≥ 18 years) admitted to the ICU, with a body mass index (BMI) ≥ 25 (kg actual BW/[height in meters]^2^). Eligible patients were those expected to require enteral nutrition starting within 48 hours after ICU admission and continuing for more than 5 days and to receive at least 800 ml of study product per day. The main exclusion criteria were a Sequential Organ Failure Assessment (SOFA) score > 12, any contraindication to tube nutrition or high protein tube feeds, and abnormalities in the gastrointestinal tract that could impair function. A complete list of inclusion and exclusion criteria is provided in Additional file 1.

### Intervention

Patients were randomized to receive either the test product or a control product for up to 28 days. The interventions stopped as soon as a patient was discharged from the ICU. Test and control products were isocaloric and contained the same blend of intact proteins, comprising animal and vegetable proteins (comprised of casein, whey, soy, and pea protein), but at different total concentrations.

The test product was a VHPF (8 g protein/100 kcal, 32 percent of energy) tube feed (1.25 kcal/ml) intended for enteral use in critically ill patients, and the control product was a commercially available SHPF (5 g protein/100 kcal, 20 percent of energy) tube feed (Nutrison Protein Plus; Nutricia, Zoetermeer, The Netherlands) used in critically ill ICU patients. More details on the macronutrient compositions can be found in Additional file 1.

The ready-to-use study products had identical packaging. Patients, investigators, and clinicians were blinded to treatment allocation.

The study products were administered through a nasogastric tube. It was recommended to start enteral feeding at a rate of 20 ml/h, increasing by 20 ml/h until the target of 25 kcal/kg IBW/d was achieved. For patients with a BMI > 30 kg/m^2^, the IBW was used to define the target volume, where IBW (kg) was calculated as: 30 × (height in meters^2^). Supplementary feeding with parenteral nutrition was allowed if necessary.

### Stratification and randomization

Permuted block randomization (randomly varying block size) to either test or control group was stratified per study center. The randomization sequence was computer-generated by a blinded statistician not involved in data collection or analysis. The randomization code was broken after database lock to enable calculations of the primary and secondary outcomes.

### Data collection

Demographic and medical information, including age, sex, height, weight, Acute Physiology and Chronic Health Evaluation II score, SOFA score, and admission category (medical, surgical, or trauma), was collected before the start of study product administration. The volume of administered study product was recorded daily until ICU discharge or day 28 of intervention. In addition, whether the patient received oral or parenteral nutrition was recorded. Reasons were recorded for each day the target intake was not met (startup period, gastrointestinal problems, medical procedures, intake via other routes, and other reasons). In case of gastrointestinal tolerance or energy intake via other routes, a specification was requested. Because of the strong decline in the number of patients in the ICU after day 10, the reported data on nutritional intake represent the first 10 days of intervention unless otherwise indicated.

Gastrointestinal tolerance parameters were recorded during the first 10 days of the intervention period, including defecation frequency, defecation consistency (according to Bristol Stool Form Scale), gastric residual volume (four times daily), and time to first defecation.

Adverse events and serious adverse events were described according to relationship to study product and underlying condition. All serious adverse events were collected, independent of the relationship with the study product. Adverse events that were not classified as serious were recorded if the event was not expected on the basis of underlying medical situation or when at least a possible causal relationship with the study product in the judgment of the treating physician was considered. The decision whether to report an adverse event could be made only by physicians who were part of the study team and specifically assigned to this task.

Adverse event recording continued until the end of the follow-up period (42 days). SOFA scores and blood samples for analyses of liver and renal function were collected at baseline, day 5, day 10, and the end of the study.

Other clinical outcome parameters were duration of ICU stay and first ventilation period, hospital stay, and mortality rates at the end of the intervention period (day 28) and at the end of follow-up (day 42).

Serum concentrations of amino acids were determined at baseline and day 5. Blood samples were collected in serum-separating tubes. After precipitation of proteins and polypeptides with perchloric acid, samples were centrifuged. The serum samples were stored at − 80 °C for analysis at a central laboratory. The content of the individual amino acids was determined in the supernatant by ultrafast liquid chromatography using a precolumn derivatization with *o*-phthaldialdehyde and fluorimetry as detection. The samples were analyzed in one batch at the end of the study.

At day 5 ± 1, urine was collected if feasible at study sites for a duration of 24 hours. Samples were analyzed at the central laboratory for total nitrogen content.

### Outcomes

The primary outcome parameter was protein intake from study product in grams per kilogram of body weight (g/kg BW) at day 5 of the intervention period. Secondary outcome parameters included protein and energy intake from study product at each day per kg BW, as a percentage of target (where energy target was defined as 25 kcal/kg BW and protein target as 1.5 g/kg BW), and as the average intake over the first 10 days of intervention and the total intervention period. Calculations of protein and energy intake were performed both per kg IBW and per kg actual body weight (ABW); IBW and ABW values are reported for the primary parameter, whereas only IBW data are reported for the secondary parameters to ensure consistency with prescribed target volumes.

The incidence of diarrhea was assessed using the daily defecation score (DDS), which is the sum of consistency scores for every evacuation per day. Diarrhea was defined as a DDS > 15 for at least 1 day or a DDS between 6 and 15 for at least 2 consecutive days (previously described in [19]). Constipation was defined as no bowel evacuation during 72 hours. The incidence of vomiting was derived from adverse event monitoring throughout the intervention period. The change in serum amino acid concentrations from baseline to day 5 and total urinary nitrogen content at day 5 were exploratory outcome parameters.

Safety assessments included patient medical history, medication use, and adverse events throughout the intervention period and at follow-up (day 42). Additional safety assessments were adverse events of special interest (vomiting and severe adverse events possibly, probably or definitely related to the study product) and parameters of hepatic function (alkaline phosphatase, alanine aminotransferase, aspartate aminotransferase, γ-glutamyltransferase, and ammonia) and renal function (creatinine, blood urea nitrogen [BUN], and cystatin C). An independent data monitoring committee (DMC) performed safety reviews after each fourth reported death of a subject.

### Statistical analysis

This study was powered to detect an effect size of 0.5 g/kg BW for protein intake at day 5 [20]. Assuming a significance level (α) of 0.05 and a two-sided effect, a sample size of 38 patients resulted in 80% power to observe a statistically significant and clinically relevant difference in protein intake. The study included six additional patients to cover possible unevaluable subjects due to missing data, early terminations, or protocol deviations.

All analyses were performed on the intention-to-treat dataset, defined as all subjects randomized. For the primary parameter, least squares (LS) means are reported. LS means provide the average of the estimated effects from the linear mixed model by assuming missing at random for missing data and including the stratification factor of center as a covariate. For other continuous outcome parameters, mean ± SD or, for skewed distributed data, median and IQR are reported unless otherwise specified. For categorical outcome parameters, count and percent are reported.

For all outcome parameters, two-sided *P* values < 0.05 were considered statistically significant, without correction for multiple testing. Analyses were performed with SAS version 9.4 software (SAS Institute Inc., Cary, NC, USA).

A repeated measures mixed model is fitted with intervention group, day, and interaction of group by day, including the stratification factor of center as fixed effects and considering day as a within-subject factor with a compound symmetry variance-covariance structure. When the normality assumption was not satisfied, nonparametric van Elteren tests (stratified Wilcoxon-Mann-Whitney test) were performed to compare the intervention groups. The robustness of the model results was checked by performing sensitivity analysis excluding some predefined outliers. Other continuous outcome parameters were analyzed using two-way analysis of variance, including the center and intervention group. Incidences were compared across the intervention groups using a chi-square test or Fisher’s exact test.

For time-to-event outcomes, Cox proportional hazards regression models were used to compare the mortality rates of the intervention groups.

## Results

### Participant flow

Between March 5, 2016, and February 23, 2017, 6423 ICU patients were prescreened for eligibility. Reasons for prescreen failures were BMI < 25 kg/m^2^ (46%), expected ICU stay < 5 days (36%), age < 18 years (1%), SOFA score > 12 (< 1%), and “other” (15%); only one patient had no information on the reason for screen failure. All 44 randomized subjects completed the study and were included in the intention-to-treat analysis.

### Baseline characteristics

The groups were well-balanced, and there were no notable differences in baseline characteristics (Table 1).

**Table 1:** Subject characteristics at baseline

	SHPF group (*n* = 22)	VHPF group (*n* = 22)
Sex, male, *n* (%)	13 (59.1%)	9 (40.9%)
Age, yr, mean (SD)	60.8 (15.2)	63.9 (13.3)
Body weight, kg, mean (SD)	91.2 (20.7)	84.9 (18.3)
BMI, kg/m^2^, mean (SD)	30.7 (8.4)	30.3 (4.1)
Admission diagnosis, *n* (%)		
Medical	9 (40.9%)	8 (36.4%)
Surgical	10 (45.5%)	11 (50.0%)
Surgical nontrauma^a^	4 (18.2%)	4 (18.2%)
Surgical trauma	6 (27.3%)	7 (31.8%)
Trauma	9 (40.9%)	10 (45.5%)
Trauma nonsurgical	3 (13.6%)	3 (13.6%)
SOFA score, median [IQR]	9 [7–11]	10 [9–11]
APACHE II score, median [IQR]	24 [18–27]	25 [21–28]
Predicted mortality, %, mean (SD)	48.4 (18.7)	52. 6 (17.7)
Adjusted predicted mortality, %, mean (SD)	38.7 (19.8)	42.7 (20.3)

*Abbreviations: BMI* Body mass index, *SOFA* Sequential Organ Failure Assessment, *APACHE II* Acute Physiology and Chronic Health Evaluation II, *SHPF* Standard high protein formula, *VHPF* Very high protein formula

^a^Surgical trauma patients were included in both the surgical and trauma subgroups of patients

### Protein intake

The primary parameter, protein intake from study product at day 5, was statistically significantly higher in the VHPF group (1.49 g/kg ABW/d; 95% CI 1.21–1.78) than in the SHPF group (0.76 g/kg ABW/d; 95% CI 0.49–1.03), with a treatment difference of 0.73 g/kg ABW/d; *P* < 0.001). Expressed per kg IBW, intake values were 1.54 g/kg IBW (95% CI 1.26–1.83) versus 0.80 g/kg IBW (95% CI 0.52–1.07), respectively, with a treatment difference of 0.74 g/kg IBW (*P* < 0.001). Descriptive statistics for protein intake at day 5 are displayed in Additional file 1.

**Fig. 1:**
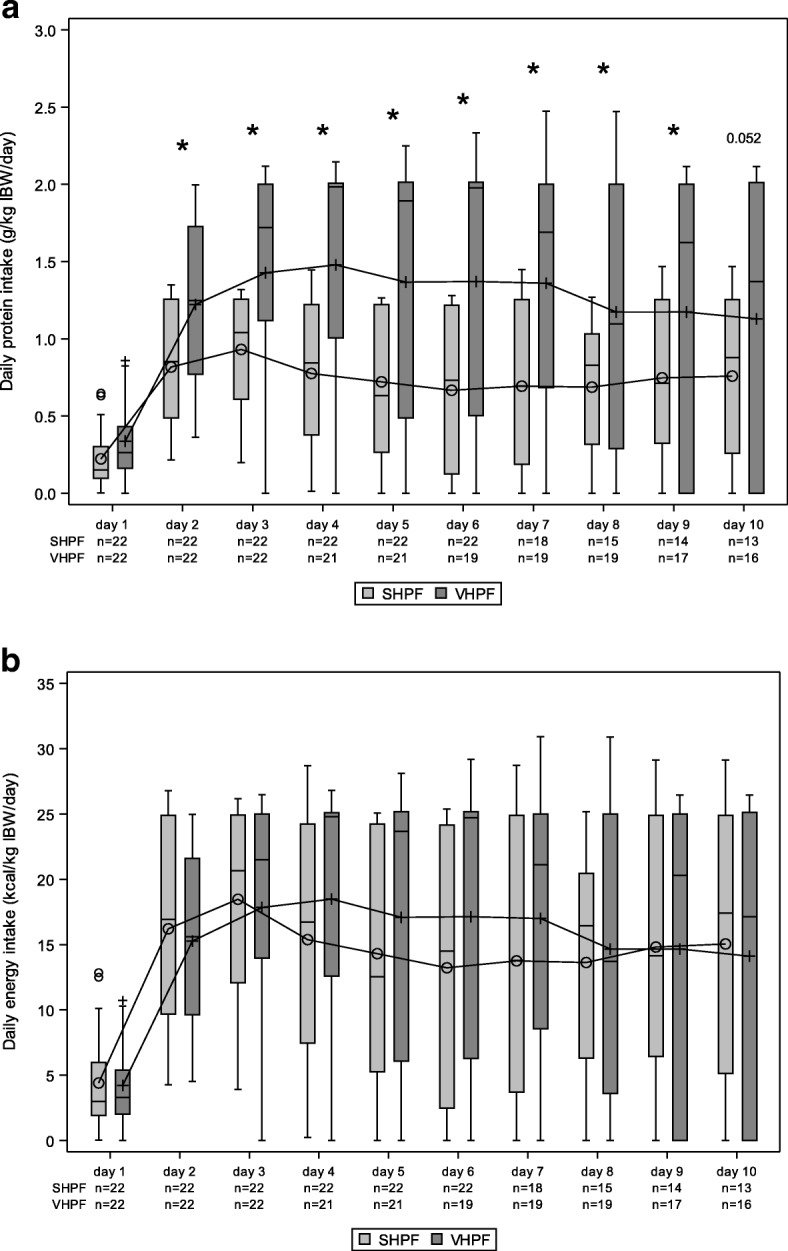
Daily protein and energy intake during the first 10 days of the intervention period. The figure shows daily protein intake (g/kg ideal body weight [IBW]/d) from study product (**a**) and daily energy intake (kcal/kg IBW/d) from study product (**b**) for the standard high-protein formula (SHPF) and very high protein formula (VHPF) groups. SHPF, *n* = 22; VHPF, *n* = 22. Box plot interpretation: 0 or + average value, −: median, rectangle bottom: quartile 1 cut point (25th percentile), rectangle upper: quartile 3 cut point (75th percentile). 0 or +: outliers more than 1.5 times IQR above quartile 3 or below quartile 1, T: highest or lowest level not being an outlier. * Statistically significant between-group differences derived from a repeated measures mixed model with intervention group, day, and interaction of intervention by day including the stratification factor center as fixed effects and considering day as a within-subject factor with a compound symmetry variance-covariance structure

Daily protein intake was statistically significantly higher in the VHPF group than in the SHPF group from day 2 to day 10. At day 10, there was a trend toward a higher intake with VHPF (*P* = 0.052) (*see* Fig. 1a).

**Fig. 2:**
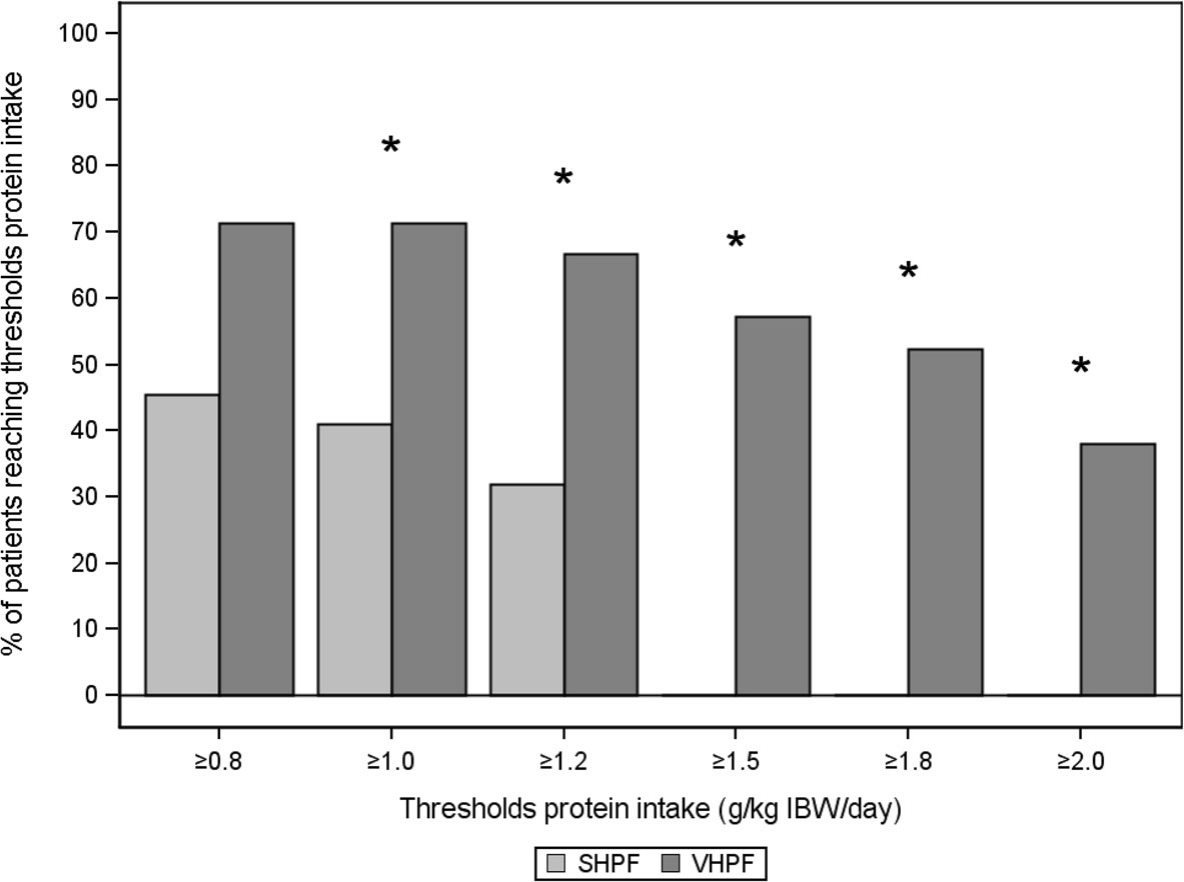
Percentage of patients reaching threshold protein intake. Percentage was calculated on the basis of number of subjects in the study on day 5 (standard high-protein formula [SHPF], *n* = 22; very high protein formula [VHPF], *n* = 21). * Statistically significant between-group differences derived by chi-square test (for ≥ 0.8, ≥ 1.0, and ≥ 1.2 g/kg ideal body weight [IBW]/d threshold protein intake) or Fisher’s exact test (for ≥ 1.5, ≥ 1.8, and ≥ 2.0 g/kg IBW/d threshold protein intake)

Figure 2 shows the percentage of subjects at day 5 reaching a protein intake above thresholds ranging from 0.8 to 2.0 g/kg IBW; the percentage of subjects achieving target intake was statistically significantly higher in the VHPF group than in the SHFP group for all targets except 0.8 g/kg IBW. The protein target of 1.5 g/kg IBW/d was reached in 57% of patients in the VHPF group but in none of the patients in the SHPF group (*P* < 0.001).

**Fig. 3:**
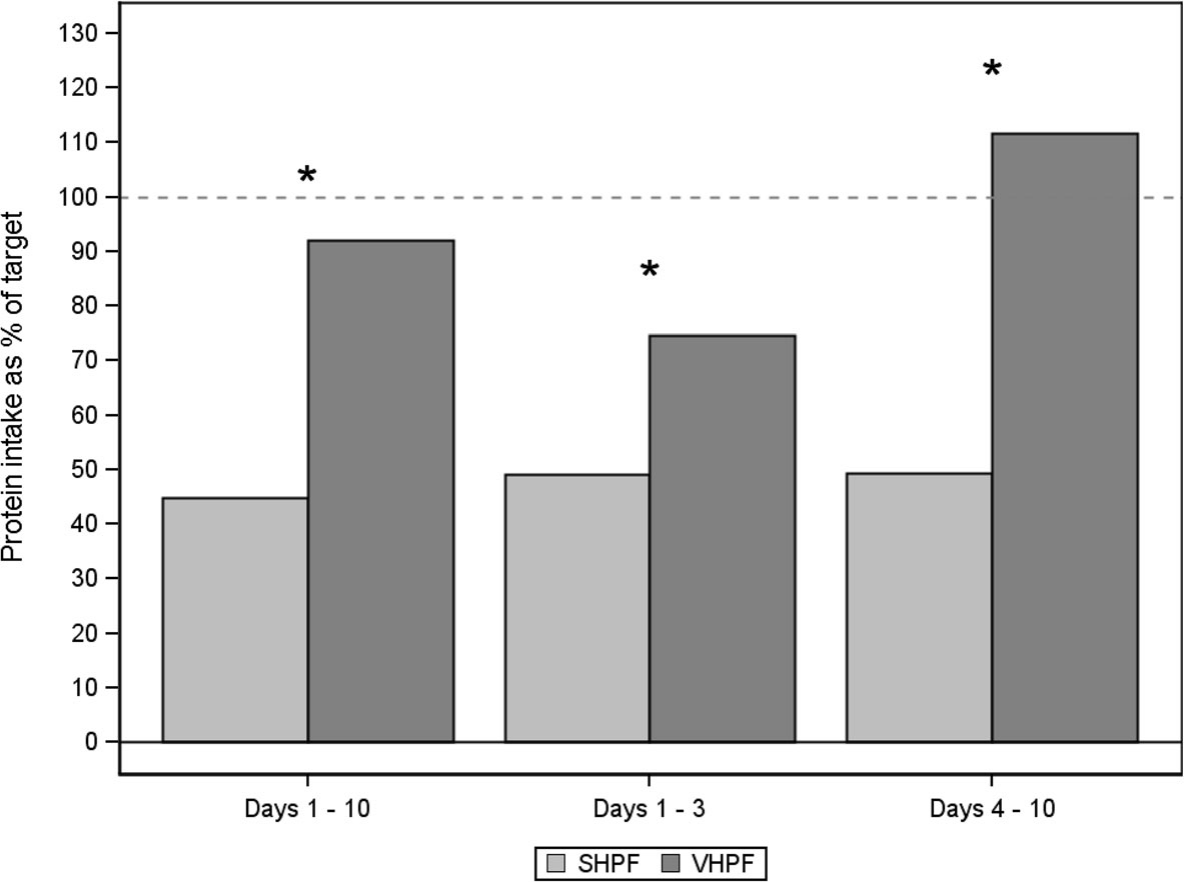
Protein intake as a percentage of target. Protein intake as percent of target for the first 10 days of the intervention period, days 1–3 and days 4–10. Standard high-protein formula (SHPF; *n* = 22) and very high protein formula (VHPF; *n* = 22) protein intake as a percentage of target is presented as median protein intake as a percentage of target (1.5 g/kg ideal body weight/d). * Statistically significant between-group difference derived by van Elteren test, stratified for center

Figure 3 shows that the intake as percent of protein target achieved was 92% (IQR 48–114%) for VHPF subjects and 45% (IQR 38–60%) for SHPF subjects (*P* = 0.007). Corresponding data were 75% (IQR 53–88%) versus 49% (IQR 26–56%), respectively (*P* = 0.009), in the startup period (days 1–3) and subsequently 112% (IQR 52–131%) versus 49% (IQR 27–70%), respectively (*P* = 0.005), from days 4 through 10. The mean cumulative protein deficit at day 10 was 292 ± 555 g in the VHPF group versus 741 ± 187 g in the SHPF group (*P* = 0.022). Protein intake averaged over all ICU days (up to and including day 28) was statistically significantly higher for the VHPF group than for the SHPF group (median 1.3 [IQR 0.7–1.9] g/kg IBW/d versus 0.7 ([IQR 0.5–0.9] g/kg IBW/d, respectively; *P* = 0.011).

### Energy intake

The average daily energy intake from study product for the first 10 days of the intervention period was 1162 ± 606 kcal in the VHPF group and 1163 ± 375 kcal in the SHPF group (*P* = 0.476). No statistically significant between-group differences in daily energy intake (kcal/kg IBW/d) were found (*see* Fig. 1b). Study product provided 69% (IQR 36–85%) versus 53% (IQR 45–71%) of the calculated caloric target (25 kcal/kg IBW/d) in the VHPF and SHPF groups, respectively. The corresponding data were 56% (IQR 40–66%) versus 58% (IQR 31–67%), respectively, in the startup period (days 1–3) and subsequently 84% (IQR 39–98%) versus 59% (IQR 32–83%), respectively, from days 4 through 10. No statistically significant differences between groups were found (*P* = 0.333).

The proportions of total ICU days when the energy intake target was not reached were 72% in the VHPF group and 84% in the SHPF group. In the first 3 days, the main reason for not reaching the target was startup period in 95% of subjects (21 of 22) in both groups, representing 53% and 55% of ICU days in the VHPF and SHPF groups, respectively. From days 4 to 10, the most frequently reported reasons for not reaching target energy intake in the VHPF and SHPF groups were medical procedures (13% versus 34% of ICU days) and energy intake via other routes (13% versus 27% of ICU days). In addition, “other reason” was reported in 21% of ICU days in both intervention groups.

Parenteral nutrition was received by zero to two patients in both study groups in each of the first 5 days of intervention. In the following days, up to six patients received parenteral nutrition because of either complete switch to parenteral feeding or as supplemental parenteral nutrition. Daily energy and protein intake from parenteral nutrition for days 1–10 is displayed in Additional file 1.

Energy intake averaged over all ICU days (up to and including day 28) showed no statistically significant difference between the VHPF and SHPF groups (median 16.6 [IQR 8.9–23.3] kcal/kg IBW/d and 14.4 [IQR 10.9–18.8] kcal/kg IBW/d, respectively; *P* = 0.729).

### Gastrointestinal tolerance and clinical outcome

Table 2 shows gastrointestinal and clinical outcome parameters. No statistically significant differences were found between groups for the incidences of diarrhea, constipation, vomiting, high gastric residual volume, or any of the clinical outcomes for the first 10-day period. The daily defecation score and the occurrence of high gastric residual volume and vomiting per day are described in Additional file 1.

**Table 2:** Gastrointestinal and clinical outcomes

		SHPF group (*n* = 22)	VHPF group (*n* = 22)	*P* value
Incidence of gastrointestinal tolerance parameters^a^				
Diarrhea	*n* (%)	11 (50.0%)	8 (36.4%)	0.361^b^
Constipation	*n* (%)	16 (72.7%)	13 (59.1%)	0.340^b^
High gastric residual volume (> 500 ml)	*n* (%)	5 (22.7%)	4 (18.2%)	0.567^c^
Vomiting	*n* (%)	6 (27.3%)	5 (22.7%)	1.000^d^
Clinical outcome parameters				
Mortality rate				
Total 28 days	*n* (%)	3 (13.6%)	2 (9.1%)	0.560^e^
Total 42 days	*n* (%)	3 (13.6%)	3 (13.6%)	0.886^e^
Hospital	*n* (%)	3 (13.6%)	2 (9.1%)	0.638^c^
ICU	*n* (%)	2 (9.1%)	1 (4.5%)	0.637^c^
Duration of ICU stay (day)	mean (SD)	18.3 (12.7)	18.4 (13.4)	0.913^f^
	95% CI	12.7–23.9	12.4–24.3	
Duration of hospital stay (day)	Mean (SD)	28.2 (13.2)	28.5 (13.3)	0.955^f^
	95% CI	22.4–34.1	22.5–34.4	
Duration of first ventilation period (day)	Mean (SD)	7.4 (5.4)	10.0 (8.7)	0.234^f^
	95% CI	5.0–9.8	6.1–13.9	
SOFA scores				
Screening	median [IQR]	9 [7–11]	10 [9–11]	0.514^g^
Day 5	Median [IQR]	6 [4–8]	6 [3–8]	0.647^g^
Day 10	Median [IQR]	5 [4–9]	4 [1–7]	0.432^g^
End of study	Median [IQR]	2 [1–3]	2 [1–3]	0.608^g^
Day 28	Median [IQR]	1 [0–2]	3 [2–3]	0.446^g^

*Abbreviations: SOFA* Sequential Organ Failure Assessment, *ICU* Intensive care unit, *SHPF* Standard high protein formula, *VHPF* Very high protein formula

^a^Incidence is defined as at least one event during the first 10 days of the intervention period

^b^*P* value derived by chi-square test

^c^*P* value derived by Cochran-Mantel-Haenszel test

^d^*P* value derived by Fisher’s exact test

^e^*P* value derived by Cox proportional hazards regression analysis with study product and site as stratification factors

^f^*P* value derived by two-way analysis of variance with treatment and center as factors. Four subjects were not discharged from the ICU at follow-up. For these subjects, ICU and hospital stays were limited to day of follow-up (day 42 ± 3 days)

^g^*P* value derived by van Elteren test (stratified for center)

Gastrointestinal intolerance was a reason for not reaching the energy intake target in 9% of ICU days (8% and 10% in the VHPF and SHPF groups, respectively). In the first 3 days, intolerance was reported as a reason for not reaching the target volume in 11 of 44 of the included subjects (3 of 22 versus 8 of 22 subjects in the VHPF and SHPF groups, respectively), and for the subsequent days, this was a reason for 11 of 44 of subjects (5 of 22 versus 6 of 22 subjects).

### Safety

The DMC performed a planned safety review after the fourth subject death. On the basis of semiblinded (recoded to X and Y) interim analysis data (serious adverse events, mortality, and adverse events of special interest), the DMC recommended continuing the study as planned.

In total, 57 adverse events were reported among 28 patients: 23 among 12 patients in the VHPF group and 34 among 16 patients in the SHPF group. There were 7 adverse events classified as gastrointestinal disorders in the VHPF group and 12 in the SHPF group. None of the adverse events was reported as related to the use of the study product in the VHPF group, whereas in the SHPF group, two adverse events were classified as possibly related and one as probably related to the use of the product. There were eight serious adverse events among five subjects in the VHPF group and seven among six subjects in the SHPF group, but none was related to use of the study products. A list of the reported adverse events per body system is displayed in Additional file 1.

Serum BUN levels at day 5 were statistically significantly higher in the VHPF group than in the SHPF group (median 15.7 [IQR 7.6–22.6]) versus 11.5 [IQR 6.8–14.4] mmol/L, respectively; *P* = 0.045). No statistically significant between-group differences were found for other laboratory safety parameters.

### Serum amino acids

**Fig. 4:**
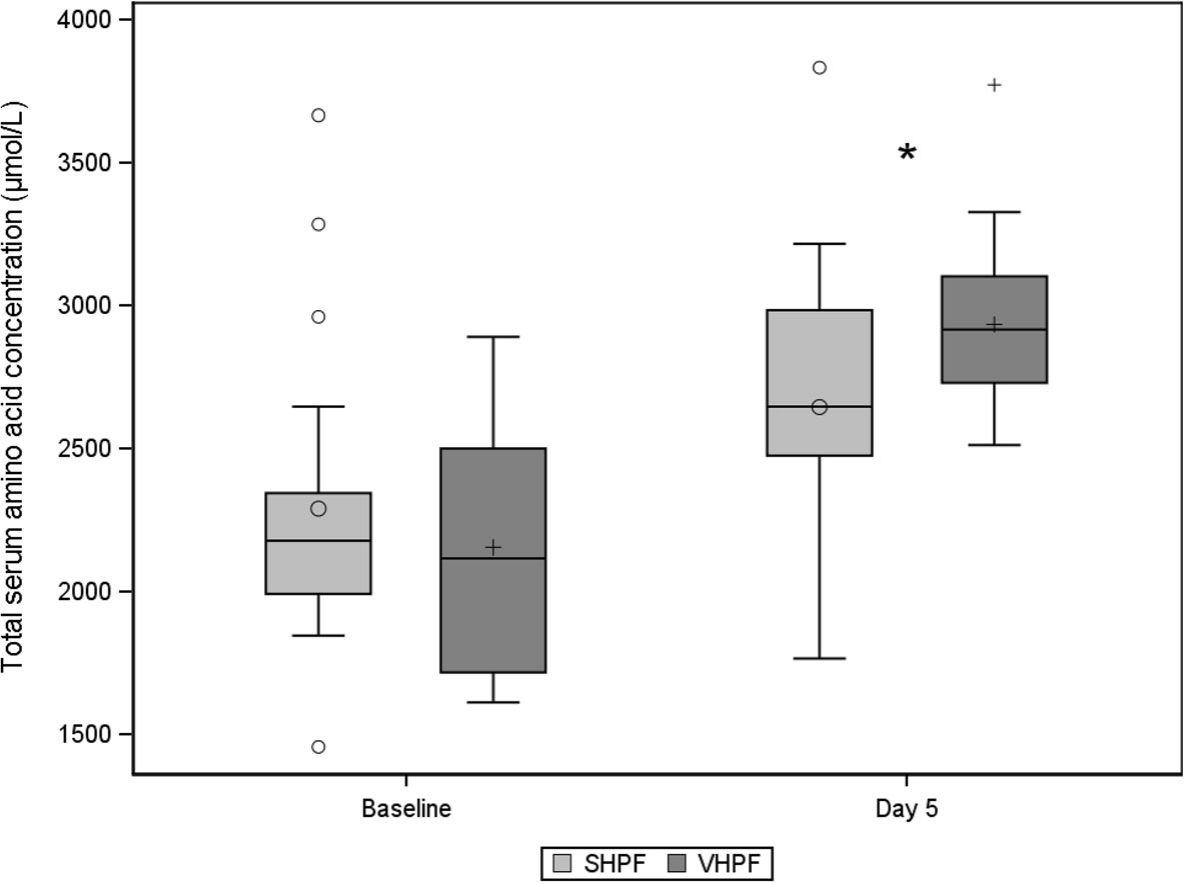
Total serum amino acid concentrations at baseline and day 5. The total serum amino acid concentration is the sum of leucine, isoleucine, valine, histidine, lysine, methionine, phenylalanine, threonine, tryptophan, alanine, arginine, asparagine, aspartic acid, citrulline, cysteine, glutamic acid, glutamine, glycine, serine, and tyrosine. Box plot interpretation: 0 or +: average value, −: median, rectangle bottom: quartile 1 cut point (25th percentile), rectangle upper: quartile 3 cut point (75th percentile). 0 or +: outliers more than 1.5 times IQR above quartile 3 or below quartile 1, T: highest or lowest level not being an outlier. * Statistically significant between-group difference derived by two-way analysis of variance with treatment, center and baseline as factors. Baseline measurements that were not within 4 hours before the start of study product administration were excluded from statistical analysis (standard high-protein formula [SHPF], *n* = 20; very high protein formula [VHPF], *n* = 18)

At baseline, the serum concentrations of total amino acids were 2154 ± 426 μmol/L in the VHPF group and 2289 ± 510 μmol/L in the SHPF group (*P* = 0.346). At day 5, serum levels were significantly increased in both groups (*P* < 0.001). The mean increase from baseline was statistically significantly higher with VHPF (835 ± 483 μmol/L) than with SHPF (408 ± 688 μmol/L; *P* = 0.031). Figure 4 shows that levels at day 5, corrected for baseline levels, were statistically significantly higher in the VHPF group than in the SHPF group (*P* = 0.031).

### Urinary nitrogen

Total urinary nitrogen was statistically significantly higher in the VHPF group than in the SHPF group (24 g [18–27] versus 18 g [14–21], respectively; *p* = 0.001).

## Discussion

To our knowledge, this is the first randomized controlled trial to demonstrate that feeding with an enteral VHPF (8 g/100 kcal) compared with an isocaloric SHPF (5 g/100 kcal) results in markedly higher protein intake without an increase in energy intake, gastrointestinal intolerance, or adverse events. These results suggest that this VHPF is suitable as first-line nutritional treatment for critically ill patients because it offers a solution for adequate protein provision according to nutritional guidelines while avoiding the risk of overfeeding. In addition, higher serum amino acid levels are reached with VHPF than with SHPF, indicating that additional proteins provided enterally are absorbed and available to serve as a substrate for protein synthesis.

Energy intake from study product was not different between the groups in this study and on average did not exceed the suggested 70% of the estimated goal of 25 kcal/kg IBW. This indicates that subjects were not unintentionally overfed by enteral administration. However, it must be noted that some patients had additional intake from parenteral nutrition.

Although recent studies have shown that increasing energy intake above 70–90% of target does not reduce mortality rates, providing protein intake close to targets has been associated with a decrease in mortality and length of ICU stay [8–10, 21, 22]. Based on these outcomes, consensus guidelines suggest that achieving protein targets is more important than achieving energy targets in the initial phase of ICU treatment [1, 11]. The median protein intake over the first 10 days was 0.7 g/kg IBW in the control group, which is in line with data from observational studies [6, 16]. The median intake of 1.4 g/kg IBW with the VHPF used in the present study confirms that a formula with a very high protein-to-energy ratio provides protein in the recommended range of 1.2–2.0 g/kg. In previous studies, protein goals could be achieved only by using additional parenteral amino acids or protein supplements [23, 24]. In contrast, in this study, the target of 1.5 g/kg IBW was reached with the VHPF in the majority of the subjects at day 5, whereas none of the subjects in the control group achieved this level. In this study, we chose to focus on a target protein intake of 1.5 g/kg BW/d to reflect most commonly used goal likely to be sufficient for the majority of ICU patients. For some patients, however, the optimal intake is believed to be around 2.0 g/kg BW/d. This level is achieved only in patients reaching the caloric target of 25 kcal/kg BW/d, which in this study, in the VHPF group, occurred in 38% of the patients at day 5. This is concordant with previous observations suggesting that the mean caloric intake is not higher than 75% of target. Additional protein supplementation may still be relevant in specific patient groups that need protein intake over 2 g/kg BW/d and low caloric intakes. Therefore, it should be noted that the new product may not be sufficient in all patients. Moreover, our study population included some patients for whom enteral nutrition appeared to be infeasible or no longer needed at the measured time point. For these patients, another strategy is required to provide sufficient dietary protein such as enteral protein or oral nutrition supplements or even parenteral amino acid supplementation. However, for the majority of ICU patients, even for those with moderate overweight, the product provides enough proteins to meet the guidelines.

The energy intake from study product was not found to be different between groups; therefore, the higher protein intake in the VHPF group can be attributed to the higher protein content of the product and not the higher volume of study product intake. To our knowledge, there is one other published study showing that protein targets can be achieved with a VHPF, although the formula was based on hydrolyzed protein [25]. It has been suggested that a predigested formula may potentially be beneficial in improving tolerance [24], but data are lacking to support this hypothesis, and nutritional guidelines recommend the use of polymeric formulas in critically ill patients [11, 26, 27].

The new VHPF was well tolerated, with no increased incidence of diarrhea, constipation, high gastric residual volumes, or vomiting versus the control product. In comparison with previous studies investigating gastrointestinal tolerance, the present study population showed similar or better tolerance outcomes overall, as evidenced by the similar incidence of high gastric residual volume [28, 29] and lower incidence of feeding interruptions due to intolerance. In the present study, intolerance was a reason for not reaching the feeding target in 25% of subjects compared with 30.5% of subjects in a large observational study by Gungabissoon et al. [28]. The subject characteristics may provide a possible explanation for the favorable outcomes on gastrointestinal tolerance in this study; for example, intolerance and high gastric residual volumes are more frequently observed in head trauma patients [30]. Moreover, both formulas used in this study are based on a protein blend (P4 protein blend comprised of whey, casein, soy, and pea) that has been shown in in vitro studies and in non-critically ill patients to be noncoagulating and emptied faster by the stomach than a standard formula based on casein [31–33].

In addition to gastric emptying, the ability of the gut to digest and absorb the dietary provided protein is an important factor determining its efficacy, especially in critically ill patients where impairments in digestion and absorption of macronutrients have been shown [34–36]. The question arises whether the gut has sufficient capacity to digest higher amounts of protein. The higher serum amino acid concentrations in the VHPF group than in the SHPF group suggest that the increased provision of protein into the small intestine leads to higher amino acid availability in the plasma. It is likely that the higher amino acid levels in the blood are due at least in part to higher appearance of amino acids derived from nutrition. However, stable isotope-labeled amino acid tracer studies are needed to investigate the effect of higher protein intake on protein synthesis and breakdown in the peripheral and central body compartments.

Nitrogen excretion in urine, measured as an exploratory parameter in this study, was significantly higher in the VHPF group. This was expected because inefficient use of dietary protein during critical illness has been observed before [37]. Nitrogen balance theoretically can be derived from the difference between nitrogen intake and excretion. However, in our study, we encountered several methodological issues limiting reliable calculations. There were considerable variations in timing of urine collection partnered with high daily variations in nutritional intake. This precludes adequate matching of protein intake and nitrogen loss for the majority of the patients, and therefore precise nitrogen balances could not be derived. Although BUN levels were significantly higher at day 5 in the VHPF group, there were no differences in plasma levels of ammonia, creatinine, or cystatin C, suggesting no differences in renal function as expressed by the glomerular filtration fraction estimated by plasma creatinine levels or cystatin C or hepatic detoxification function with the high levels of protein intake provided [38]. This is in line with results from the study by Doig et al. showing no effects of intravenous amino acid provision on the duration of renal dysfunction [39].

A particular strength of this study is that the study products were isocaloric and based on the same protein source, which allows the effect of the amount of protein on gastrointestinal tolerance to be studied without confounding influences from other product differences. Another strength is that the study design allowed enteral feeding according to nutritional guidelines to be compared with a control group reflecting current practice.

The specific study population of overweight and obese patients was included because a high protein-to-energy ratio was considered especially important in this group. Consequently, these results are not representative of the total ICU population, which is a limitation of this study. Another weakness is that the total protein and energy intake, including intake from oral nutrition, was not recorded; therefore, the reported data underestimate the total nutritional intake. In addition, it should be taken into account that the plasma amino acids found at day 5 were somewhat influenced by protein intake from oral and parenteral nutrition. Some subjects with very low intake received oral nutrition. For example, at day 5, reported intake from enteral nutrition was zero or very low in three patients in each group; however, according to investigators’ notes, intake from oral nutrition was substantial in some cases (complete meals up to 1800 kcal/d). Total energy intake is a missing component in most studies investigating this topic because recording this parameter is time-consuming [40]. In future studies, it would be of interest to record calories and protein from oral intake or other sources to better understand the contribution to the total intake.

The study protocol specified a relatively rapid startup period, but in practice, the progression toward nutritional targets was somewhat slower than planned. In the VHPF group, 75% of protein target was reached in the first 3 days, and 112% of protein target was reached in days 4 to 10, whereas caloric adequacy was 56% and 84%, respectively. Although the lower caloric intake was not intentional, it is in line with the current advice for conservative caloric delivery, supported by studies indicating that optimal targets in the acute phase may be around 70–90% of energy expenditure [9, 10].

## Conclusions

The widely supported consensus is that nutritional provision, with special focus on protein, is of pivotal importance during and after critical illness. The present study shows markedly higher protein intake without an increase in energy intake with a polymeric VHPF, indicating that provision of protein according to nutritional guidelines is feasible with an enteral nutrition product based on intact proteins. The results imply that a VHPF is suitable as first-line enteral feeding treatment in ICU patients.

## Additional file

Additional file 1: Full list of inclusion and exclusion criteria, product composition of control (SHPF) and test (VHPF) products, descriptive statistics on protein intake at day 5, intake from parenteral nutrition, gastrointestinal parameters per day, number (*k*) and incidence (*n*) of (S)AEs per body system. (DOCX 45 kb)

## Abbreviations

ABW: Actual body weight
APACHE: Acute Physiology and Chronic Health Evaluation
BMI: Body mass index
BUN: Blood urea nitrogen
BW: Body weight
DDS: Daily defecation score
DMC: Data monitoring committee
IBW: Ideal body weight
ICU: Intensive care unit
LS means: Least squares means
SHPF: Standard high protein formula
SOFA: Sequential Organ Failure Assessment
VHPF: Very high protein formula
