# Supplementary material for: Very high intact-protein formula successfully provides protein intake according to nutritional recommendations in overweight critically ill patients: a double-blind randomized trial
Source: Crit Care. 2018 Jun 12;22:156. doi: 10.1186/s13054-018-2070-5 (PMC5998555; doi:10.1186/s13054-018-2070-5)
Supplement: Supplementary file 1 — Full list of inclusion and exclusion criteria, product composition of control (SHPF) and test (VHPF) products, descriptive statistics on protein intake at day 5, intake from parenteral nutrition, gastrointestinal parameters per day, number (k) and incidence (n) of (S)AEs per body system. (DOCX 45 kb) [file 13054_2018_2070_MOESM1_ESM.docx]

Full list of in- and exclusion criteria

*Inclusion criteria*

1. Age ≥ 18 years
2. ICU stay and indication for tube feed via nasogastric tube
3. Expected to require study product for at least 5 days on the ICU
4. BMI ≥ 25.0 kg/m^2^
5. Expected to receive a target of at least 800 ml of tube feed per day
6. Start of study product administration within 48 hours after ICU admission
7. Written informed consent of subject or written informed consent of legal representative

|  | SHPF | | VHPF | |
| --- | --- | --- | --- | --- |
|  | Per 100 ml | % of energy | Per 100 ml | % of energy |
| Energy | 125 kcal |  | 125 kcal |  |
| Protein  -whey  -casein  -pea  -soy | 6.3 g  2.2 g  1.6 g  1.3 g  1.3 g | 20% | 10 g  3.5 g  2.5 g  2.0 g  2.0 g | 32% |
| Carbohydrate | 14.2 g | 45% | 10.3 | 33% |
| Fat | 4.9 g | 35% | 4.9 g | 35% |
| Fiber | - | - | - | - |

*Exclusion criteria*

1. Requiring other specific tube feed for medical reason
2. Having any contra-indication to receive tube feed such as severe shock, presence of partial or complete mechanical bowel obstruction, or intestinal ischemia or infarction
3. Having any contra indication to receive higher protein tube feeds, such as patients with refractory hypotension, overwhelming sepsis, or serious liver disease
4. History of allergy or intolerance to the study product components (test or control product), such as allergy for cow’s milk protein, soy or pea protein, and patients with galactosaemia
5. Abnormalities in GI tract which may impact GI function, such as short bowel syndrome - defined as entire length of small bowel totaling 122 centimeters or less-, Ulcerative Colitis or Crohn’s disease or any form of enterostomy
6. GI tract, abdominal or bariatric surgery within 72 hours before start intake study product or expected in the next 5 days after start study product intake
7. History of chronic pancreatitis or acute pancreatitis
8. Expected to need parenteral feeding
9. Expected to need protein supplementation other than study product
10. SOFA score >12 from admission to the ICU until 24 hours after admission or until randomisation in case of randomisation before 24 hours after admission
11. Being pregnant
12. Participating in another clinical intervention trial

Table S1: Product composition of control (SHPF) and test (VHPF) product

Table S2: Descriptive statistics protein intake at day 5

|  |  | SHPF (N = 22) | VHPF (N = 22) | p-value^1^ |
| --- | --- | --- | --- | --- |
| g/kg actual body weight | Mean (SD)  Median (Q1-Q3) | 0.68 (0.47)  0.6 (0.3-1.2) | 1.32 (0.80)  1.6 (0.4-2.0) |  |
|  | LS mean (95% CI) | 0.76 (0.49, 1.03) | 1.49 (1.21, 1.78) | <0.001 |
| g/kg ideal body weight^2^ | Mean (SD)  Median (Q1-Q3) | 0.72 (0.47)  0.6 (0.3-1.2) | 1.37 (0.82)  1.9 (0.5-2.0) |  |
|  | LS mean (95% CI) | 0.80 (0.52, 1.07) | 1.54 (1.26, 1.83) | <0.001 |

^1^ Based on a repeated measures mixed model adjusting for treatment, day and treatment by day interaction and the stratification factor, center, using a Compound Symmetry (CS) variance-covariance structure

^2^For patients with a BMI of >30 kg/m2, the ideal body weight (IBW; 30 * (height (m) * height (m)) was used for the calculations).

Table S3: Intake from parenteral nutrition

^1^Displayed data are mean (SD) intake from PN from patients receiving PN at each specific day

| Days |  | SHPF (N=22) | |  | VHPF (N=22) | |
| --- | --- | --- | --- | --- | --- | --- |
|  | n (%) | Energy (kcal) ^1^ | Protein (g)^1^ | n | Energy (kcal) ^1^ | Protein (g) ^1^ |
| 1 | 0 (0%) | - | - | 0 (0%) | - | - |
| 2 | 1 (5%) | 514 | 27 | 1 (5%) | 514 | 27 |
| 3 | 1 (5%) | 1038 | 55 | 2 (9%) | 1156 (545) | 61 (29) |
| 4 | 1 (5%) | 1027 | 55 | 2 (9%) | 1412 (182) | 75 (10) |
| 5 | 1 (5%) | 600 | 27 | 2 (9%) | 1487 (76) | 79 (4) |
| 6 | 3 (14%) | 1268 (149) | 64 (2) | 3 (14%) | 984 (519) | 52 (28) |
| 7 | 4 (18%) | 1218 (557) | 63 (29) | 2 (9%) | 1541 (0) | 82 (0) |
| 8 | 5 (23%) | 1517 (228) | 79 (15) | 2 (9%) | 835 (817) | 44 (43) |
| 9 | 6 (27%) | 1476 (376) | 77 (22) | 2 (9%) | 749 (151) | 40 (8) |
| 10 | 5 (23%) | 1459 (670) | 76 (37) | 1 (5%) | 1027 | 55 |

Table S4: Gastrointestinal parameters per day

|  | SHPF | | | | | VHPF | | |
| --- | --- | --- | --- | --- | --- | --- | --- | --- |
| Days | DDS^1^  (median, Q1-Q3) | Incidence of GRV^2^ >500 ml (n (%)) | | Incidence of vomiting (n(%)) | | DDS^1^  (median, Q1-Q3) | Incidence of GRV^2^ >500 ml (n (%)) | Incidence of vomiting (n(%)) |
| 1-10 | 2.3 (0.2-5.1) | | 5 (22.7%) | | 6 (27.3%) | 2.6 (1.0-3.9) | 4 (18.2%) | 5 (22.7%) |
| 1 | 0 (0-0) | | 0 (0.0%) | | 0 (0.0%) | 0 (0-0) | 1 (4.5%) | 0 (0.0%) |
| 2 | 0 (0-0) | | 0 (0.0%) | | 2 (9.1%) | 0 (0-0) | 0 (0.0%) | 1 (4.5%) |
| 3 | 0 (0-3) | | 3 (13.6%) | | 0 (0.0%) | 0 (0-6) | 0 (0.0%) | 1 (4.5%) |
| 4 | 0 (0-2) | | 2 (9.1%) | | 1 (4.5%) | 0 (0-5) | 2 (9.5%) | 1 (4.5%) |
| 5 | 1 (0-7) | | 1 (4.5%) | | 1 (4.5%) | 1 (0-5) | 1 (4.8%) | 1 (4.5%) |
| 6 | 0 (0-8) | | 2 (9.1%) | | 0 (0.0%) | 0 (0-5) | 1 (5.3%) | 1 (4.5%) |
| 7 | 0 (0-3) | | 2 (11.1%) | | 0 (0.0%) | 3 (0-6)* | 1 (5.3%) | 1 (4.5%) |
| 8 | 0 (0-2) | | 1 (6.7%) | | 0 (0.0%) | 3 (0-5) | 0 (0.0%) | 0 (0.0%) |
| 9 | 0 (0-0) | | 3 (21.4%) | | 1 (4.5%) | 0 (0-5) | 1 (5.3%) | 0 (0.0%) |
| 10 | 0 (0-3) | | 1 (7.7%) | | 0 (0.0%) | 4 (1-7)* | 0 (0.0%) | 0 (0.0%) |

^1^daily defecation score

^2^gastric residual volume

* higher DDS in VHPF than SHPF p<0.05

Table S5: Number (k) and incidence (n) of (S)AE’s per body system

|  | *Total (N = 44)* | | | *SHPF (N = 22)* | | | *VHPF (N = 22)* | | |  |
| --- | --- | --- | --- | --- | --- | --- | --- | --- | --- | --- |
|  | *k* | *n* | *%* | *k* | *n* | *%* | *k* | *n* | *%* |  |
| **Any Body System** | | | | | | | | | | |
| Any event | 57 | 28 | 63.6% | 34 | 16 | 72.7% | 23 | 12 | 54.5% |  |
| **Cardiac disorders** | | | | | | | | | | |
| Atrial fibrillation | 1 | 1 | 2.3% | 0 | 0 | 0.0% | 1 | 1 | 4.5% |  |
| Bradycardia | 1 | 1 | 2.3% | 1 | 1 | 4.5% | 0 | 0 | 0.0% |  |
| Cardiac arrest | 2 | 2 | 4.5% | 1 | 1 | 4.5% | 1 | 1 | 4.5% |  |
| **Endocrine disorders** | | | | | | | | | | |
| Diabetes insipidus | 1 | 1 | 2.3% | 1 | 1 | 4.5% | 0 | 0 | 0.0% |  |
| **Gastrointestinal disorders** | | | | | | | | | | |
| Abdominal pain upper | 1 | 1 | 2.3% | 0 | 0 | 0.0% | 1 | 1 | 4.5% |  |
| Constipation | 2 | 2 | 4.5% | 2 | 2 | 9.1% | 0 | 0 | 0.0% |  |
| Ileus | 1 | 1 | 2.3% | 1 | 1 | 4.5% | 0 | 0 | 0.0% |  |
| Impaired gastric emptying | 2 | 2 | 4.5% | 2 | 2 | 9.1% | 0 | 0 | 0.0% |  |
| Large intestinal haemorrhage | 1 | 1 | 2.3% | 1 | 1 | 4.5% | 0 | 0 | 0.0% |  |
| Vomiting | 12 | 11 | 25.0% | 6 | 6 | 27.3% | 6 | 5 | 22.7% |  |
| **General disorders and administration site conditions** | | | | | | | | | | |
| Pyrexia | 1 | 1 | 2.3% | 1 | 1 | 4.5% | 0 | 0 | 0.0% |  |
| **Hepatobiliary disorders** | | | | | | | | | | |
| Cholestasis | 1 | 1 | 2.3% | 1 | 1 | 4.5% | 0 | 0 | 0.0% |  |
| **Infections and infestations** | | | | | | | | | | |
| Sepsis | 1 | 1 | 2.3% | 0 | 0 | 0.0% | 1 | 1 | 4.5% |  |
| **Injury, poisoning and procedural complications** | | | | | | | | | | |
| Colon injury | 1 | 1 | 2.3% | 0 | 0 | 0.0% | 1 | 1 | 4.5% |  |
| Procedural vomiting | 10 | 10 | 22.7% | 5 | 5 | 22.7% | 5 | 5 | 22.7% |  |
| **Investigations** | | | | | | | | | | |
| Liver function test abnormal | 1 | 1 | 2.3% | 1 | 1 | 4.5% | 0 | 0 | 0.0% |  |
| **Metabolism and nutrition disorders** | | | | | | | | | | |
| Cell death | 1 | 1 | 2.3% | 1 | 1 | 4.5% | 0 | 0 | 0.0% |  |
| Fluid overload | 1 | 1 | 2.3% | 1 | 1 | 4.5% | 0 | 0 | 0.0% |  |
| Hypermagnesaemia | 1 | 1 | 2.3% | 0 | 0 | 0.0% | 1 | 1 | 4.5% |  |
| Hypernatraemia | 1 | 1 | 2.3% | 0 | 0 | 0.0% | 1 | 1 | 4.5% |  |
| Hyperphosphataemia | 1 | 1 | 2.3% | 0 | 0 | 0.0% | 1 | 1 | 4.5% |  |
| Hypokalaemia | 1 | 1 | 2.3% | 1 | 1 | 4.5% | 0 | 0 | 0.0% |  |
| Hypophosphataemia | 1 | 1 | 2.3% | 1 | 1 | 4.5% | 0 | 0 | 0.0% |  |
| **Nervous system disorders** | | | | | | | | | | |
| Brain injury | 1 | 1 | 2.3% | 1 | 1 | 4.5% | 0 | 0 | 0.0% |  |
| Encephalopathy | 1 | 1 | 2.3% | 1 | 1 | 4.5% | 0 | 0 | 0.0% |  |
| Transient ischaemic attack | 1 | 1 | 2.3% | 0 | 0 | 0.0% | 1 | 1 | 4.5% |  |
| **Respiratory, thoracic and mediastinal disorders** | | | | | | | | | | |
| Pneumonia aspiration | 1 | 1 | 2.3% | 1 | 1 | 4.5% | 0 | 0 | 0.0% |  |
| Respiratory distress | 2 | 2 | 4.5% | 1 | 1 | 4.5% | 1 | 1 | 4.5% |  |
| Respiratory failure | 1 | 1 | 2.3% | 1 | 1 | 4.5% | 0 | 0 | 0.0% |  |
| **Surgical and medical procedures** | | | | | | | | | | |
| Ventricular drainage | 2 | 1 | 2.3% | 2 | 1 | 4.5% | 0 | 0 | 0.0% |  |
| **Vascular disorders** | | | | | | | | | | |
| Inferior vena cava perforation | 1 | 1 | 2.3% | 0 | 0 | 0.0% | 1 | 1 | 4.5% |  |
| Shock haemorrhagic | 1 | 1 | 2.3% | 0 | 0 | 0.0% | 1 | 1 | 4.5% |  |
